# Sexual orientation and gender identity documentation at an academic movement disorders neurology clinic

**DOI:** 10.1016/j.prdoa.2022.100164

**Published:** 2022-09-09

**Authors:** Roshni Abee Patel, Glenn Stebbins, Natalie Witek

**Affiliations:** a Rush University Medical Center, Department of Neurological Sciences, Section of Parkinson’s Disease and Movement Disorders, 1725 W Harrison St, Ste 755, Chicago, IL 60612, United States; b Jesse Brown VA Medical Center, Neurology Service, 820 S Damen Ave, Chicago, IL 60612, United States

Approximately 5.6% of adults identify as lesbian, gay, bisexual, or transgender (LGBT) [1], yet very little is known about the neurologic health of these individuals, and there are few published reports regarding LGBT patients with Parkinson’s disease or other movement disorders [2], [3]. Limited evidence suggests that sexual and gender minority (SGM) patients may experience inequities in health care due to discrimination and social and economic marginalization [2]. However, we cannot identify such disparities without first ensuring systematic collection of sexual orientation (SO) and gender identity (GI) data in electronic health records (EHR) [4]. In 2015, the Centers for Medicare and Medicaid Services required EHR software certified for Meaningful Use to include SO and GI fields. Subsequent analysis of over 25 million patients from 1367 health centers revealed that SO and GI data were reported in only 23% and 37% of patients, respectively [5]. Factors which may limit SO or GI (SOGI) data collection include clinician misinterpretation that patients do not want to answer these questions, inability of EHR to accommodate SOGI fields, and lack of best practices and training for systematic SOGI data collection [5]. Rush University Medical Center (RUMC) has enabled collection of SOGI in the EHR since 2016. It can be added or changed by a provider during a clinical encounter, or by the patient through an online web portal. Additional information about the SOGI collection process at Rush are provided (Supplementary Document). In this study, we sought to characterize the patterns and practices of SOGI data collection in our movement disorders neurology clinic.

All patients with encounters at the movement disorders neurology clinic at RUMC within a 4-week period in January 2021 were included in this cross-sectional study. Data were collected from the EHR including age, legal sex, sexual orientation, gender identity, race, ethnicity, primary movement disorders diagnosis, visit type (i.e. in-person vs telemedicine; new consultation vs return visit), number of outpatient encounters at Rush (with any clinical provider, not just neurology) within the previous year, language preference (English vs non-English), and patient web portal activation (which requires each patient to create an account username and password to establish access). Primary outcome was frequency of SOGI documentation. Sample size was not calculated due to the exploratory nature of this study. Binary logistic regression was used to identify factors associated with SOGI data collection. An anonymous one-time survey developed by the authors was administered to the clinic staff in June 2021 to determine knowledge and practices pertaining to SOGI data collection.

Nine-hundred and eighty-five unique patients were seen in movement disorders clinic during a 4-week period in January 2021. There was no missing data. Mean age was 65.0 years (SD 15.2), race was 77.1% white, ethnicity was 10.1% Hispanic, and legal sex was 49% female and 51% male. The most common diagnoses were parkinsonism and dystonia. Seven percent of patients were non-English speaking, and 89.7% had an active online patient portal. Telemedicine visits accounted for 42.8% of the encounters, and patients had a mean of 6.2 (SD 6.3) outpatient clinical encounters in the previous year (Table 1).

**Table 1:** Baseline characteristics of movement disorders clinic patients seen during 4-week period, frequency of SOGI documentation, and factors associated with SOGI documentation.

Characteristics	All patients (n = 985)	Factors associated with SOGI documentation, OR (95% CI)	p-value^a^
Age, mean (SD), years	65.0 (15.2)	1.01 (0.77–1.27)	0.26
Race, No. (%)		0.77 (0.59–1.01)	0.06
White	759 (77.1)		
Black	76 (7.2)		
Other	150 (15.2)		
Ethnicity, No. (%)		0.79 (0.52–1.21)	0.29
Hispanic	99 (10.1)		
Sex assigned at birth, No. (%)		0.99 (0.77–1.27)	0.91
Male	504 (51.2)		
Female	481 (48.8)		
Non-English speaking, No. (%)	65 (6.6)	0.62 (0.37–1.05)	0.08
Online patient portal activated, No. (%)	884 (89.7)	**11.0 (5.3**–**22.9)**	**<0.0001**
Primary diagnosis, No. (%)		0.97 (0.91–1.03)	0.33
Parkinsonism	617 (62.6)		
Dystonia	119 (12.1)		
Tremor	75 (7.6)		
Tics	37 (3.8)		
Huntington’s disease	35 (3.6)		
Other	102 (10.3)		
Visit Type, No. (%)		0.84 (0.63–1.13)	0.25
New	94 (9.5)		
Return	891 (90.5)		
Telemedicine	422 (42.8)	**2.15 (1.67**–**2.79)**	**<0.0001**
Number of outpatient clinical encounters in the last year, mean (SD)	6.2 (6.3)	**1.08 (1.05**–**1.11)**	**<0.0001**
Gender identity, No. (%)			
Not available	557 (56.6)		
Male	220 (22.3)		
Female	205 (20.8)		
Transgender or nonbinary	2 (<1)		
Prefer not to disclose	1 (<1)		
Sexual orientation, No. (%)			
Not available	662 (67.2)		
Straight	292 (29.6)		
Gay or lesbian	18 (1.8)		
Prefer not to disclose	13 (1.3)		

b Bolded p-values are <0.05 and considered statistically significant.

Primary outcome – frequency of SOGI documentation in the EHR– was 44.4% (438 of 985). SO was documented in 428 patients (43.4%) and GI was identified in 323 patients (32.8%). Of the 438 patients with SOGI documentation, 19 (4.3%) identified as sexual or gender minority. Factors associated with SOGI documentation included telemedicine encounter (OR 11.0, 95% CI 5.3–22.9), active online patient portal (OR 2.15, 95%CI 1.67–2.79), and increased number of outpatient clinical encounters within the previous year (OR 1.08, 95%CI 1.05–1.11). Age, race, ethnicity, legal sex, non-English speaking language preference, visit type, and primary movement disorders diagnosis were not associated with SOGI documentation (Table 1).

Among movement disorders clinic staff, 32 out of 50 completed a survey regarding SOGI documentation practices. Only 7 respondents (21.9%) knew how to input SOGI data into a patient’s EHR, and a majority (26, or 81.3%) responded “never” when asked how often they documented SOGI information for patients within the past year. When asked about the best way to collect SOGI data, 26 (81.3%) thought it should be entered by the patient either through electronic patient portal or through a patient intake form, and 17 (53.1%) thought it should be entered by the medical assistant during the rooming process (Supplementary Table).

We found that frequency of SOGI documentation at a tertiary movement disorders clinic was comparable to reports from the literature [5], but still quite low overall. Though method of how SOGI data were entered into the EHR was not available in our study, most providers reported poor knowledge of and experience with documenting SOGI, suggesting that the majority of data was entered directly by the patient via the online portal. This presumption is also supported by our finding that telemedicine encounter, activation of online patient portal, and increased number of outpatient clinic encounters were associated with SOGI documentation. Our results are consistent with other studies that suggest SOGI data collection may be most effective when collected via nonverbal self-report [6]. Importantly, age was not associated with SOGI data collection, refuting the presumption that older individuals may not want to disclose SOGI status. These results reinforce the feasibility and importance of collecting SOGI information from older adults, a pivotal step in understanding the unique health needs and disparities faced by this vulnerable and historically “invisible” population [7]. Though race and non-English speaking language preference were not associated with SOGI collection, it is possible that the study was under-powered to detect this, highlighting the importance of obtaining SOGI data in culturally appropriate ways.

Although this was a single-center study at a tertiary specialty clinic, these findings are generalizable to those caring for elderly patients or patients with chronic neurological disorders, though we recognize that differences in SOGI collection processes, as well as cultural and language differences may limit this. Future studies should investigate interventions to increase SOGI documentation, such as those that incorporate non-verbal self-reporting and/or electronic reporting by the patient. Additional efforts should be taken to train clinical staff about obtaining SOGI data in culturally appropriate ways and normalizing this process using resources like the “Do Ask, Do Tell” toolkit [8]. Improving SOGI data collection in neurology will enhance our understanding of outcomes and disparities amongst LGBT patients who have historically been hidden.

## CRediT authorship contribution statement

**Roshni Abee Patel:** Conceptualization, Methodology, Formal acquisition, Formal analysis, Investigation, Writing – original draft. **Glenn Stebbins:** Methodology, Writing – review & editing, Formal analysis. **Natalie Witeka:** Methodology, Supervision, Writing – review & editing.

## Declaration of Competing Interest

The authors declare that they have no known competing financial interests or personal relationships that could have appeared to influence the work reported in this paper.
