# Supplementary material for: Sexual orientation and gender identity documentation at an academic movement disorders neurology clinic
Source: Clin Park Relat Disord. 2022 Sep 9;7:100164. doi: 10.1016/j.prdoa.2022.100164 (PMC9483721; doi:10.1016/j.prdoa.2022.100164)
Supplement: Supplementary data 1 [file mmc1.docx]

Supplementary Table. Survey of movement disorders staff regarding SOGI documentation.

| **Survey Question** | **Responses (n=32), No (%)** |
| --- | --- |
| What is your primary role in clinic? |  |
| Clinical staff (e.g. medical doctor, registered nurse, psychologist, advanced practice provider, medical assistant) | 23 (71.9) |
| Research or Administrative staff | 9 (28.1) |
| Do you know how to input SOGI data into a patient’s electronic medical record? |  |
| Yes | 7 (21.9) |
| No | 14 (43.8) |
| Unsure/never thought about it | 11 (34.4) |
| In the last year, how often have you documented a patient SOGI in the electronic medical record? |  |
| Never | 26 (81.3) |
| A few times (less than 10% of patients) | 5 (15.6) |
| Sometimes (11-50% of patients) | 1 (3.1) |
| Frequently (over 51% of patients) | 0 |
| In your opinion, what is the best way for SOGI data to be added to a patient’s chart? (Choose all that apply) |  |
| By patient- either electronically through patient portal, or through patient intake form which is later transferred into electronic record by staff. | 26 (81.3) |
| By the medical assistant during the rooming process. | 17 (53.1) |
| By the provider by the clinical provider. | 10 (31.3) |
| Other (elaborate in the space provided)  *“patient should input into the IPAD when they check in”*  *“I add as free-text in notes, but not sure where the official field is located”* | 2 (6.3) |

**Description of the SOGI collection process at Rush**

Rush University Medical Center (RUMC) has enabled collection of SOGI in the EHR since 2016. The following fields can be completed in the “Demographics” portion of the chart, but they are optional (not required) as opposed to the Name, Date of Birth and Legal sex fields, which are required.

SOGI fields and options:

- Sexual orientation: Straight (not lesbian or gay), Bisexual, Gay, Lesbian, Something else, Don’t know, Choose not to disclose
- Gender Identity: Female, Male, Transgender Female/Male-to-Female, Transgender Male/Female-to-Male, Non-binary/Neither exclusively man or woman, Other, Choose not to disclose

SOGI data can be added or changed by a clinical provider during a clinical encounter (including medical assistant, nurse, physician or advanced practice provider), or by the patient through online web portal. There is no institution-wide policy or training regarding SOGI data collection; each clinic and department may have their own individual practice. In our movement disorders clinic, SOGI data collection is not part of the routine intake process for patients, and we found that providers overall had low knowledge of and experience with adding SOGI information in the EHR (Supplementary Table).
